# The implicit association between masculinity and criminal organizations

**DOI:** 10.1038/s41598-025-03984-8

**Published:** 2025-06-06

**Authors:** Giovanni A. Travaglino, Maddalena Marini

**Affiliations:** 1 Department of Law and Criminology, School of Law and Social Sciences, Royal Holloway, University of London, Egham, TW20 0EX UK; 2 Department of Psychology, University of Campania “Luigi Vanvitelli”, Viale Ellittico, 31, 81100 Caserta, Italy

**Keywords:** Masculinity, SC-IAT, Criminal organizations, Legitimacy, Implicit associations, Human behaviour, Psychology

## Abstract

Criminal groups, such as Italian criminal organizations, exert governance over communities. According to Intra-Cultural Appropriation Theory (ICAT), these groups can gain a degree of legitimacy by strategically appropriating masculinity values. Specifically, by portraying themselves as embodying masculinity, criminal organizations are evaluated more positively by individuals who endorse masculine honor ideologies. An untested assumption of this process is that individuals ascribe masculine qualities to criminal groups. In two studies (*N*_*tot*_ = 310), we employed the Single Category Implicit Association Test to investigate whether individuals implicitly associated the categories of ‘male’ (Study 1) and ‘masculinity’ (Study 2) with criminal organizations as opposed to the state. Additionally, in Study 2, we tested whether this implicit association moderated the relationship between individuals’ endorsement of masculine honor ideology and their attitudes toward criminal organizations. The findings supported the hypothesis that individuals implicitly attributed masculinity-related concepts to criminal organizations. Study 2 further showed that the positive link between endorsement of masculine honor ideology and legitimizing attitudes towards criminal organizations was stronger when individuals also held a stronger implicit association between masculinity and criminal organizations.

Criminal groups such as mafias, large gangs, and other criminal networks may exert power and authority over territories^1–4^. These groups regulate other criminals’ activities and manage illegal enterprises^5^. Notably, their influence extends beyond the criminal underworld to the broader non-criminal population. In some areas, criminal organizations engage in governance-related functions that are typically within the domain of the state, including imposing norms, mediating conflicts, levying taxes, and providing protection^4,6–10^. A prime example of this dynamic is seen in Italian mafias, which can displace legal institutions in the areas where they operate.

Criminal groups’ ability to control territories and exert authority over communities enables them to thrive, with substantial negative implications for democracy and citizens’ rights. However, like other forms of authority^11^, their power needs to be grounded in some form of legitimacy to be effective. This raises the crucial research question of what are the processes that allow criminal groups to gain legitimacy within the community. Addressing this question is important not only to advance our knowledge of how these groups operate within the social context but also to provide insights into how social power and authority can be wielded outside legal and institutional channels.

Recent research has shown that the endorsement of masculinity-related values predicts individuals’ legitimization of criminal organizations^12^. Embedded in this research is the assumption that criminal organizations successfully portray themselves as embodying ideals of masculinity, exhibiting features and traits that resonate with certain individuals’ values^13^. However, this assumption has yet to be empirically tested. In the present research, we extended previous work by investigating whether individuals ascribe qualities of masculinity to criminal organizations at an implicit level. Specifically, across two studies conducted in Italy, we examined whether individuals implicitly associated the general and abstract category of male (Study 1) and the more specific category of masculinity (Study 2) with criminal organizations as opposed to state. Additionally, in Study 2, we explored a potential implication of the implicit association for individuals’ legitimization of criminal groups.

## Intracultural appropriation theory, masculinity, and criminal organizations

Social power refers to the capacity to affect others’ thoughts and behaviors. This capacity may rely on coercion – the use of material or social resources to enforce compliance^11^ Coercive power can produce immediate and visible effects, but it is often unstable because it provokes resistance and opposition^11^. In contrast, power rooted in legitimacy, typically referred to as authority, is perceived as appropriate and consistent with shared norms or standards. Legitimacy reduces the psychological and material costs of compliance and lowers the risk of defiance, making it a more effective and enduring foundation for social power^11,14^.

Legal authorities derive their legitimacy from adhering to fair and just procedures^15^, which foster self-regulation in followers, promote voluntary obedience, and decrease the need for constant surveillance. When such authorities are perceived as legitimate, individuals are more likely to internalize their directives and comply because they believe it is the right thing to do. But how do groups that are criminal, violent, and dangerous gain the capacity to exert authority? These groups may rely on coercive power, including violence and intimidation, to impose control^6^ However, coercion alone is insufficient for maintaining long-term influence. To sustain authority, criminal organizations must also cultivate some form of legitimacy to secure compliance, minimize resistance, and embed within communities.

This capacity to exert authority is particularly paradoxical, as the practices of criminal organizations often violate norms of fairness and justice. For example, paying mafias money in exchange for protection does not guarantee that they will provide the services they promise^16^. Yet, despite these contradictions, such groups continue to wield influence, prompting important questions about the mechanisms through which they cultivate legitimacy within the communities where they exert control.

Intra-Cultural Appropriation Theory (ICAT) explains criminal groups’ capacity to exert authority by positing that these groups strategically exploit values and cultural codes shared in the community^4^. ICAT identifies a process of ‘strategic exploitation’, whereby criminal groups present themselves as the embodiment of shared cultural values, using adherence to these values to justify their actions and gain legitimacy. Through their practices and discourses, criminal groups reinforce the values’ centrality in society, further consolidating their standing.

ICAT has been used to examine how criminal groups appropriate specific cultural values – particularly those related to masculinity. Broadly defined, masculinity refers to the socially consensual way of being an adult male in a given society^17,18^. Masculinity may be expressed through specific qualities, including strength, courage, and virility^19^. In regions such as the Mediterranean,^20^ these specific masculine qualities are integral to a broader cultural framework of honor, which prescribes the behaviours expected of ‘real men’. Within cultures of honor, male strength, violence, and other expressions of masculinity are not only tolerated but may be admired, especially when used to defend one’s public image and property from encroachment^21–23^. These expectations reflect what Connell termed hegemonic masculinity^24^: a culturally idealized form of masculinity that legitimizes male dominance and sustains existing hierarchies of power^25^. Hegemonic masculinity privileges aggression, control, and toughness, while marginalizing alternative masculinities, thereby reinforcing the notion that legitimacy – whether social or institutional – can be tied to the enactment of dominant masculine traits.

Criminal groups strictly adhere to and portray themselves as embodiments of honor and, more specifically, the masculine qualities valorized in this cultural framework^4^. For instance, mafia members refer to themselves as ‘made men’ or ‘men of honor’. They are highly prone to risk^26^ and use violence symbolically to punish insults against their reputation or threats to their virility. They emphasize patriarchal norms in their dealings with other affiliates and the broader community. This strategic focus on masculinity is not unique to Italian mafias; it also characterizes the practices and discourses of other criminal groups. Members of North^27–29^ or South American gangs^30^, the Japanese Yakuza^31^ and cliques in Brazil, Russia and Germany^32^ similarly adhere to strict codes of masculinity. These recurring dynamics suggest that masculine ideals may serve as a cultural resource through which criminal groups in diverse contexts seek to integrate themselves into the communities they control.

Criminal groups’ adherence to masculinity has generally been understood in light of their propensity for violence^33–35^. This view aligns with several theoretical models that examine how the endorsement of masculinity-related values fosters aggressive behavior^36,37,18^. For example, O’Dea et al.’s Masculinity-Based Model of Aggressive Retaliation in Society (MARS) proposes that the endorsement of values related to masculinity and honor interact with broader social norms, role expectations, and biological predispositions to facilitate violence and criminal conduct^38^ Together with similar models, MARS helps explain why and how masculinity plays a central role in shaping violent behavior within criminal groups.

Complementing these perspectives, ICAT proposes that symbolic displays of masculinity can also regulate and shape how criminal groups engage with communities. Specifically, ICAT suggests that masculinity values can function as an ideological framework that lends legitimacy to the actions of such groups^39^. This framework allows their authority to be not only tolerated but also perceived as appropriate within the social context. By strategically aligning their behavior with shared masculine norms, criminal organizations can legitimize their activities and secure obedience from the population. Thus, masculinity serves not merely as a conduit for aggression, but as a symbolic resource that helps criminal groups establish authority.

Consistent with this conceptualization of masculinity, empirical research conducted in Italy has shown that individuals’ endorsement of ideologies of masculine honor is associated with a more favorable view of criminal groups^12^. Specifically, individuals who endorsed these ideologies of masculine honor tended to report higher legitimising attitudes and lower intentions to oppose mafias. Recent longitudinal evidence further suggests the existence of reciprocal, within-person relationships between the masculine honor ideology and legitimizing attitudes, reinforcing the notion that criminal groups’ practices may also strengthen the relevance of these values for individuals^40^.

A crucial assumption of this research is that individuals perceive criminal groups as embodying masculine-related qualities. However, this assumption has so far remained untested, partly due to the challenges of studying people’s beliefs about criminal groups. In contexts such as Italy, institutional norms demand that criminal organizations be condemned unequivocally^41^. As a result, describing these groups in ways that might be perceived as condoning the mafia phenomenon is problematic. Such norms could render self-report measures less effective in capturing the *specific* traits (as opposed to more generic attitudes) individuals attribute to criminal organizations^42^. To address this issue, in the present research, we investigated the association between masculinity and criminal organizations using implicit measures.

## Implicit measures of association

Individuals’ answers to self-report measures largely depend on intentional choices, which may be susceptible to biases and concerns about social desirability^42^. In contrast, implicit measures infer individuals’ beliefs and attitudes through tasks that are more difficult to manipulate intentionally and less likely to be influenced by conscious decisions^43–45^. These ‘indirect’ instruments are, therefore, better suited to accessing psychological content that individuals may be unwilling or reluctant to report openly.

To examine the association between masculinity and criminal organizations, in this research, we employed a variant of the Implicit Association Test (IAT)^46,47^, specifically the Single Category IAT (SC-IAT)^48^. The IAT is a widely used instrument to assess mental associations. It has been applied in numerous research fields and disciplines, including social psychology^49,50^, cognitive psychology^51^, developmental psychology^52,53^, clinical psychology^54,55^, and neuroscience^50,56–59^. Similarly to the IAT, the SC-IAT relies on measures of response latencies and classification errors as indicators of the strength of implicit associations between concepts^60^.

For example, in a traditional IAT that assesses associations between categories like ‘*flowers*’ and ‘*insects’* and attributes such as *‘pleasant’* and ‘*unpleasant*’, individuals are asked to categorize images of or words related to flowers (e.g., rose and tulip) and insects (e.g., bee and ant), and pleasant (e.g., love and peace) or unpleasant (e.g., bad and fear) words in two different sorting conditions. In one condition, participants press a specific key to categorize pictures of flowers or pleasant words, while pressing a different key to categorize pictures of insects or unpleasant words. In the second condition, they press one key to categorize flowers or unpleasant words and another key for insects or pleasant words. The IAT is based on the principle that categorizing becomes easier (quicker and with fewer errors) when two mentally related concepts share the same response key. For instance, if an individual has a mental association between flowers and pleasantness, they will respond faster and with fewer mistakes when the same key is used for both. The SC-IAT’s structure is very similar to that of the IAT, with the only difference being that a single category (or attribute) is employed together with two attributes (or categories).

Both the IAT and the SC-IAT are relative measures of association^61^, meaning that they require the inclusion of a second category (or attribute) to provide a comparison. The task’s outcomes are determined by the relative strength of the associations between pairs of concepts. In the context of this research, we aim to examine the association between the concepts of masculinity and criminal organizations. According to ICAT, this association plays a crucial role in enabling criminal organizations to gain legitimacy and exert governance^4^. To provide a meaningful comparison, we included the category of ‘state’ in the SC-IAT. This allows us to measure how strongly individuals associate masculine qualities with criminal organizations in contrast to the state, with which these organizations sometimes compete for authority.

## Overview of the studies

The objective of this research was to investigate whether the concept of masculinity is more closely associated with criminal organizations than with the state. We employed implicit methods to test the hypothesis that participants associate the concept of masculinity more strongly with that of ‘criminal organizations. This prediction stems from ICAT’s assumption that criminal organizations portray themselves as embodiments of masculinity values to legitimise their activities^4^, as well as empirical evidence showing that individuals’ endorsement of masculine honor values is linked to the legitimization of these groups^12^.

We examined our hypothesis across two studies conducted in Italy, a country characterised by the strong presence of mafia-type groups. In Study 1, we explored the association between the idea of male, operationalized at an abstract level using male names, and criminal organizations. In Study 2, we focused on the more specific concept of masculinity, which we operationalized using terms such as strength and courage (see Table 1 for the complete list), reflecting consensual standards and qualities that define what it means to be a ‘real man’ in honor cultures^17,18,21^.

**Table 1:** Labels and stimuli used in the SC-IAT across studies.

Studies	Labels	Stimuli
Study 1	Male	Alessandro, Lorenzo, Gabriele, Riccardo, Tommaso
	Criminal organizations	Camorra, ‘Ndrangheta, Mafioso, Camorrista, ‘Ndranghetista
	State	Governo, Costituzione, Legge, Parlamento, Presidente
Study 2	Masculinity	Virilità, Forza, Potenza, Protezione, Coraggio
	Criminal organizations	Camorra, ‘Ndrangheta, Mafioso, Camorrista, ‘Ndranghetista
	State	Governo, Costituzione, Legge, Parlamento, Presidente

The translation for the Italian stimuli is: Governo = Government, Costituzione = Constitution, Legge = Law, Parlamento = Parliament, Presidente = President; Virilità = Virility, Forza = Strenght, Potenza = Power, Protezione = Protection, Coraggio = Courage.

In Study 2, we also explored a potential implication of ascribing masculinity to criminal organizations. Specifically, we examined whether a stronger implicit association between these two concepts moderated the relationship between participants’ endorsement of the masculine honor ideology and their explicit legitimization of criminal organizations. Following ICAT, we hypothesized that this relationship would be stronger among participants who displayed a stronger implicit association between criminal organizations and masculinity.

## Study 1

No previous research has investigated whether participants attribute masculinity to criminal organizations using implicit methods. Thus, the goal of Study 1 was to establish, for the first time, whether this implicit link exists by examining the abstract category of male.

### Methods

#### Participants and procedures

One-hundred-fifty-five participants (*M*_age_ = 21.88, *SD* = 7.05, 48.39% women) were recruited via the Prolific Web site, which was selected based on prior research indicating that it provides high-quality and reliable data for psychological studies^62^. Participants were born in various Italian regions, including Campania (15% of the sample), Lazio (17%) and Piemonte (11%). Each of the remaining regions accounted for less than 7% of the sample. We predetermined a sample size of *N* = 155, which enabled us to detect a small effect size, *d* = 0.22, at 80% power (α = 0.05), according to the G*Power software (v. 3.1.9.2)^63^. Participants were eligible to take part in the study if they were 18 years or older, were born in Italy, and reported Italian as their primary language.

Participants were invited to participate in “A study on the categorization of words and concepts”. Materials were in Italian. Participants completed a SC-IAT which measured the strength of implicit association between the attribute male and the social categories criminal organization and state. After completing the SC-IAT, participants provided demographic information (gender, age, and Italian region where they were born). They were then debriefed about the scope of the research, thanked, and compensated for their time. The study sessions lasted about ten minutes on average.

The SC-IAT followed the standard procedure described by Karpinski and Steinman^48^. Participants classified stimuli by pressing one of two response keys on their keyboard in two different sorting conditions. In one sorting condition, participants classified “male” and “criminal organization” stimuli by using the same response key and a different key to sort “state” stimuli. In another sorting condition, participants switched to classify “male” and “state” stimuli by using the same response key and a different key to sort “criminal organization” stimuli. Each sorting condition consisted of 24 practice trials immediately followed by 72 test trials. Sorting conditions were randomized across participants, and stimuli were presented one at a time in the center of the computer screen. Male Italian first names (e.g., Alessandro and Lorenzo) were used as stimuli for the attribute “male”. In contrast, different words referring to criminal organizations (e.g., Camorra and ‘Ndrangheta) and state (e.g., Government and Parliament) were used as stimuli for the social categories “criminal organizations” and “state”, respectively. Stimuli for the attribute “male” and the categories “criminal organization” and “state” are presented in Table 1. The study was approved by the University of Kent’s ethics committee and was conducted in accordance with the guidelines of the British Psychological Society and the American Psychological Association. Informed consent was obtained from all participants in the study.

### Results

SC-IAT scores were computed using the algorithm described by Greenwald, et al.^64^. Specifically, we divided the difference in mean response between the two sorting conditions by the participant’s latency standard deviation inclusive of the two conditions. Responses faster than 350 milliseconds and slower than 10,000 milliseconds were removed, and errors were replaced with the mean of the correct responses in that response block plus a 600-millisecond penalty.

Positive scores indicated an association between the concepts of “male” and “criminal organizations”. Scores could range from + 2 to -2, with zero indicating neutrality or no difference in the associations of either category (“criminal organization” or “state”) with the attribute “male”. Using standard criteria, participants whose results indicated careless participation were excluded from our data (4.91%). Specifically, we removed participants who (1) made more than 30% errors or (2) were faster than 350 ms on more than 10% of the critical trials.

To test whether participants showed a significant implicit association between the concepts of “male” and “criminal organizations”, we computed a one-sample t-test against zero, which indicates no association. The analyses revealed a medium-sized effect, indicating a significant implicit association between the two concepts, *M* = 0.14, *SD* = 0.24, *t*(154) = 7.385, *p* < 0.001, Cohen’s *d* = 0.59, 95% *C.I.* [0.42, 0.76].

### Discussion

In Study 1, we tested the hypothesis that participants associate the male concept with criminal organizations more strongly than with the state. Supporting this hypothesis, participants responded faster and more accurately in the SC-IAT condition where they categorized male names with terms related to criminal organizations, as opposed to terms related to the state. This finding, obtained using abstract stimuli (i.e., male first names), suggests the existence of a basic-level association between the concepts.

However, it is important to acknowledge a potential confound related to the methodology of this study. In the Italian context, the most recognizable members of criminal organizations are predominantly male^65^, which may have influenced participants’ responses. As such, the observed association may reflect a general identification of mafia groups with men, rather than male-related qualities. We address this limitation in Study 2, where we directly examine attributes of masculinity rather than relying on gendered name stimuli.

## Study 2

In Study 2, we extended the results of the previous study by investigating whether attributes of masculinity, operationalized using terms such as strength and virility, were more closely associated with the category “criminal organization” than “state.” Additionally, we tested the hypothesis derived from ICAT^4^ that participants’ endorsement of the masculine honor ideology would be more strongly associated with the legitimization of criminal organizations when they also held a stronger implicit link between masculinity and criminal organizations.

### Methods

#### Participants and procedures

One-hundred-fifty-five participants (*M*_*age*_ = 22.79, *SD* = 7.74, 49% women) were recruited via the Prolific website. Participants reported they were born in various Italian regions, including Lazio (14% of the sample), Lombardia (13%) and Piemonte (12%). Each of the remaining regions accounted for less than 9% of the sample. We predetermined a sample size of *N* = 155. This sample size enabled us to detect a small effect size, *d* = 0.22, at 80% power (α = 0.05). As in Study 1, participants were eligible to take part in the study if they were 18 years or older, were born in Italy, and reported Italian as their primary language.

Participants completed a SC-IAT measure similar to that used in Study 1. However, In Study 2, we assessed the associations between the social categories of criminal oganization and state with the specific attribute masculinity rather than the more generic male. Words referring to masculinity (e.g., virility and strength) were used as stimuli (see Table 1 for a full list). After completing the SC-IAT, participants completed explicit self-report measures assessing their endorsement of the masculine honor ideology and the legitimization of criminal organizations. They were asked to provide some demographic information (gender, age, and Italian region where they were born), after which they were debriefed, thanked, and compensated for their time. On average, study sessions lasted ten minutes. The study was approved by the Department of Psychology, University of Kent’s Ethics Committee, and was conducted in accordance with the guidelines of the British Psychological Society and the American Psychological Association. Informed consent was obtained from all participants in the study.

#### Explicit measures

##### Honor ideology for manhood

 As in Drury and Travaglino^13^, individuals’ endorsement of the masculine honor ideology was measured using ten items drawn from Barnes et al.’s scale^21^. We used the Italian translation of the scale employed in prior research^13^. Items tapped into individuals’ agreement about the qualities that should define a ‘real man’ (e.g., “A real man will never back down from a fight”) and the use of male violence to respond to insults (e.g., “A man has the right to act with physical aggression toward another man who openly flirts with his wife”; 1 = *strongly disagree*, 7 = *strongly agree*). Scale items are not self-descriptive and measure the perceived suitability of male violence and the endorsement of specific male qualities that define a “real man”. The items can, therefore, be completed by all sexes. Items were internally reliable (α = 0.86) and were averaged to produce a single score of the endorsement of the masculine honor ideology.

##### Legitimizing attitudes

 Individuals’ legitimization of criminal organizations was measured using four items adapted from previous research^12^. The items – originally developed in Italian – were: “Some of Mafias’ aims are legitimate”, ‘Some actions of Mafias may have positive direct or indirect consequences for the territory”, “Actions of Mafias deserve respect”, ‘Actions of Mafias deserve admiration’ (*1* = *strongly disagree*, *7* = *strongly agree*). The items were internally consistent (α = .80) and were averaged in a unique score of legitimizing attitudes.

### Results

#### Implicit associations

SC-IAT scores were computed as in Study 1. Positive scores indicated an association between “masculinity” and “criminal organizations”. Scores could range from + 2 to -2, with zero indicating neutrality or no difference in the association of either category (“criminal organization” or “state”) with the attribute “masculinity”. Using standard criteria, 1.90% of data were excluded as indicative of careless participation. A one-sample t-test against zero revealed a strong implicit association between masculinity and criminal organizations, *M* = 0.22, *SD* = 0.27, *t*(154) = 10.150, *p* < 0.001, Cohen’s *d* = 0.82, 95% *C.I.* [0.63, 1.00].

#### Testing the interaction between masculine honor ideology and SC-IAT scores on legitimizing attitudes

In line with previous research, participants’ endorsement of the masculine honor ideology was positively correlated with legitimizing attitudes towards criminal organizations. The SC-IAT scores were not significantly associated with the Masculine Honor Ideology (correlation coefficients are summarized in Table 2).

**Table 2:** Correlation among variables in study 2.

Variables	1	2	3	4	5
1 SC-IAT Score	–				
2 Masculine Honor Ideology	0.09				
3 Legitimizing Attitudes	0.19	0.39***			
4 Sex	0.22*	0.44***	0.22*		
5 Age	0.18	0.08	− 0.03	− 0.01	-

*p < 0.05, ***p < 0.001.

We tested a regression model in which the SC-IAT moderated the relationship between masculine honor and individuals’ legitimising attitudes towards criminal organizations. Age and gender were entered into the model to control for the effects. Note that excluding these covariates did not alter the results. The overall model was significant, *F*(5, 145) = 8.89, *p* < 0.001, and explained 24% of the variance in the outcome. Controlling for the SC-IAT score, the masculine honor ideology positively predicted participants’ legitimising attitudes, β = 0.28, *t* = 4.66, *p* < 0.001. Controlling for the masculine ideology, the SC-IAT scores were also positively associated with participants’ legitimising attitudes, β = 0.18, *t* = 3.06, *p* = 0.003. The hypothesized interaction between masculine honor ideology and the SC-IAT scores on participants’ legitimising attitudes was significant, β = 0.17, *t* = 3.068, *p* = 0.003.

**Fig. 1:**
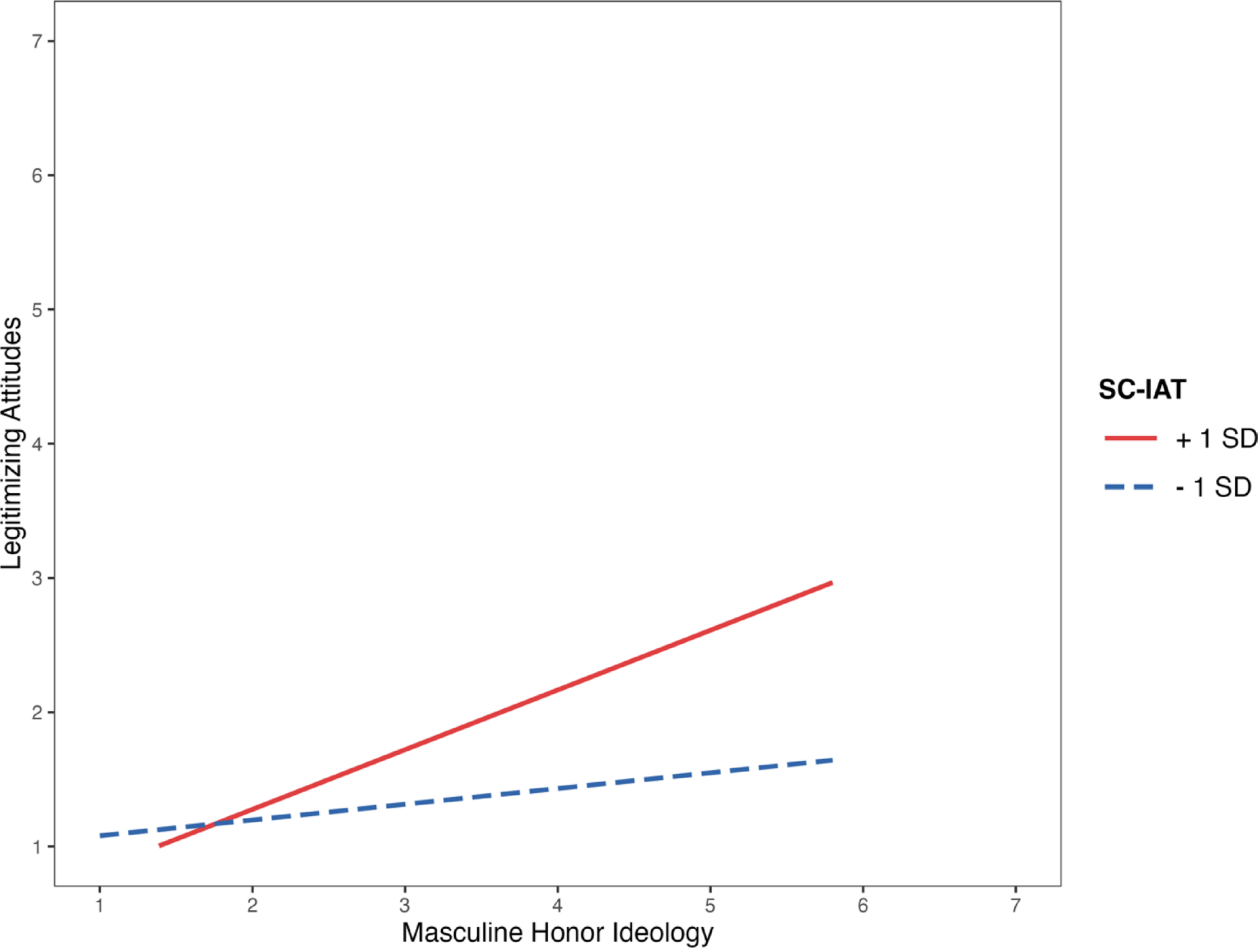
Association between masculine honor and legitimizing attitudes towards criminal organizations at different levels of the SC-IAT score. Note. Gender and age were covariates in the model.

We proceeded to inspect the significance of the simple slopes of masculine honor at ± 1 *SD* of SC-IAT scores (see Fig. 1). In line with ICAT, participants’ endorsement of the masculine honor ideology was significantly linked to the legitimization of criminal organizations only when participants attributed “masculinity” more strongly to criminal organizations than the state. Specifically, at + 1 SD of SC-IAT scores, the association between masculine honor and criminal legitimization was strong and statistically significant (β = 0.44, *t* = 5.52, *p* < 0.001), indicating that a one standard deviation increase in masculine honor was associated with a 0.44 standard deviation increase in the legitimization of criminal organizations. In contrast, at − 1 SD of SC-IAT scores, the association was weaker and not statistically significant (β = 0.12, *t* = 1.45, *p* = 0.15), suggesting that when masculinity was less strongly associated with criminal organizations, endorsement of masculine honor was not reliably related to the perceived legitimacy of these groups.

### Discussion

In Study 2, we predicted and found that participants ascribed features of masculinity more strongly to criminal organizations than the state. The present study provided the first empirical demonstration that individuals implicitly associate attributes such as ‘strength’, ‘protection’, and ‘courage’ with criminal organizations. This finding extended the results from Study 1, which used a more abstract operationalization of masculinity (i.e., male names), reinforcing the notion that criminal organizations are perceived as embodying specific masculine qualities at both an abstract and specific level.

In this study, we also addressed a key implication of the implicit association between masculinity and criminal organizations. Specifically, we examined whether the strength of this association would moderate the relationship between participants’ endorsement of the masculine honor ideology and their legitimizing attitudes toward these groups. Our hypothesis was based on ICAT, which posits that criminal organizations portray themselves as the embodiment of masculinity to gain legitimacy. This suggests that individuals who hold stronger implicit associations between masculinity and criminal organizations would also exhibit a stronger link between masculine honor endorsement and legitimizing attitudes.

Results showed that participants’ endorsement of the masculine honor ideology predicted the legitimization of criminal organizations. Consistent with our hypothesis, this association was significant only when participants held a stronger implicit association between criminal organizations and masculinity. This finding highlights the critical role that masculinity values play in shaping and regulating the relationship between criminal organizations and the community. The process tested in the present study may underpin criminal organizations’ public assertions of masculinity in their discourses and practices, helping explain why these groups are particularly attuned to detecting and punishing deviations from masculinity norms among affiliates, as well as to identifying and responding to threats to their masculine reputation that may arise from within the broader community. The results from this study are consistent with the notion that, by being perceived as embodying masculine qualities, criminal organizations may enhance their ability to maintain authority. It is therefore essential for these groups to preserve their reputation as embodiments of masculinity in the eyes of the community.

## General discussion

Although criminal organizations engage in unfair and violent behavior, they are able to replace the state, assume some of its functions, and exert authority over territories and communities^5,6,9,16^. It is, therefore, crucial to investigate the mechanisms that enable criminal organizations to gain relatively stronger legitimacy among the public. While prior research has examined how individuals’ endorsement of masculine ideology relates to their views of criminal organizations, less is known about the specific qualities people implicitly attribute to these groups^4^. In the present research, we investigated whether participants attributed qualities of masculinity to criminal organizations. Specifically, we employed the SC-IAT to examine whether participants associated attributes of male and masculinity more strongly with criminal organizations than with the state. This research was grounded in Intra-Cultural Appropriation Theory, which posits that these groups gain legitimacy by portraying themselves as embodying masculinity values shared in the communities^4^.

In Study 1, we established an implicit association between the attribute ‘male’ and criminal organizations. In this study, the concept of male was operationalized using neutral male first names, indicating a basic link between the two concepts. In Study 2, we extended these findings by employing more specific attributes of masculinity, such as ‘strength’ and ‘courage’, which are highly valued in cultural areas where honor is a predominant value^20,21^. In Study 2, participants attributed masculinity more strongly to criminal organizations than to the state. Importantly, we also predicted and found that the strength of this attribution moderated the relationship between participants’ explicit endorsement of the masculine honor ideology and their legitimizing attitudes towards criminal organizations. This relationship was significant only for participants who more strongly associated masculinity with criminal organizations at the implicit level.

The findings are consistent with the idea that individuals ascribe masculine qualities to criminal organizations, at least relatively to the state. These results contribute to theorizing about the function of masculinity-related values in the context of criminal groups. Previous research in this area has primarily emphasized the role of these values in fostering violence or structuring status relationships within criminal groups^34^. Our findings extend this understanding by demonstrating that masculinity-related values also play a crucial role in how criminal organizations are perceived and legitimized within the broader community.

Evidence from the present research suggests that, consistent with ICAT, the shared endorsement of masculinity values at an implicit level may contribute to establishing positive relationships between criminal groups and the community. ICAT posits that cultural values play a key role in individuals’ perceptions of extra-legal or illegal groups seeking to exert influence. For these values to serve as a basis for legitimization, however, the group must successfully present itself as conforming to socially endorsed standards, a process referred to as *intracultural appropriation*. Evidence of such conformity may emerge when individuals implicitly associate the group with the very values it seeks to appropriate. Consistent with ICAT, in the context of Italian organized crime, when criminal organizations are implicitly associated with masculinity, they receive greater legitimization among individuals who endorse the masculine honor ideology. The present findings help illuminate the mechanisms through which such groups enhance their capacity to exert influence – and, more broadly, how culture, and particularly shared value systems, can contribute to the legitimization of criminal authority within local communities.

## Limitations and future research directions

A limitation of these studies is that the SC-IAT provides only a relative measure of association. Our studies compared criminal organizations with the state, as we were specifically interested in understanding the contrast between these groups and legal authorities. However, the extent to which these implicit associations generalize to other categories remains an open empirical question. Future research should explore whether similar associations between criminal organizations and masculinity emerge when compared to other relevant categories.

Additionally, our studies focused on a specific cultural context – Italy – where mafia-type organizations are embedded in the social fabric. The findings may not be fully generalizable to other countries or regions characterized by different criminal or cultural dynamics. As discussed earlier, according to ICAT, for cultural values to become a source of legitimacy, there must be a perceived fit between the way the group represents itself and the values shared within the community^4^. This framework implies that groups in other sociocultural contexts may appeal to different values to secure legitimacy. For instance, in individualistic Western societies, some groups acting illegally may draw on values such as autonomy or anti-elitism in addition to (or rather than) masculinity^66^. In non-Western contexts, legitimacy might instead be grounded in notions of collective protection and community defense.

Moreover, even the content of what constitutes masculinity is likely to vary across cultural settings. For instance, in the Italian context, masculinity may emphasize dominance, strength, and the willingness to use violence – traits embedded in honor cultures. In other settings, masculinity may be more strongly tied to the capacity to provide financially and to demonstrate economic independence. In such contexts, criminal groups may strategically emphasize these aspects of masculinity to recruit members and establish credibility^30^ Future research should investigate how masculinity is culturally constructed and appropriated by criminal groups across different regions, and how these constructions intersect with broader systems of values, power, and legitimacy.

Another limitation of the present research is that its correlational design does not permit causal inferences. Although our findings are consistent with the theoretical predictions of ICAT, the associations observed between masculinity-related values, implicit associations, and legitimization of criminal organizations cannot be interpreted as evidence of causality. Future research employing experimental or longitudinal designs is needed to establish the directionality of these effects and to better understand the causal mechanisms underlying these relationships.

An additional area for future research concerns the systematic study of the processes through which criminal groups signal their conformity to masculine-related qualities to the community. News reports indicate that affiliates of criminal groups use social media to publish photos with weapons or post about their defiance of rules and authorities^67^. Additionally, research has documented criminal groups’ use of popular songs, graffiti, and banners to display messages to the public^68–70^. These channels, along with public acts of violence and the strategic use of high-profile murders, may serve as a means to communicate adherence to masculinity. Understanding the communication strategies employed by criminal groups could help shed light on how criminal organizations cultivate their image and sustain their influence.

Finally, further research is needed to examine the implications of implicit attributions of masculinity in other contexts. For instance, there is some evidence that individuals who are particularly concerned with their masculine status are more likely to support political leaders who project strength and toughness^25,71^. Future studies should investigate how masculinity is implicitly attributed to various social actors – such as state institutions or political figures – and how these attributions interact with masculine ideology across cultural and political settings. Such research could offer valuable insights into how implicit gendered schemas may shape public perceptions of authority more in general (Fig. 1).

## Conclusions

How criminal groups can gain the legitimacy necessary to exert authority and influence is a crucial research question that has received surprisingly little attention. In this article, we examined whether individuals implicitly attribute qualities of manhood to criminal organizations and explored the implications of such attributions for legitimizing these groups. Our findings highlight the importance of considering the role that values of masculinity play in shaping and regulating the relationships between criminal groups and the community. Beyond their role in fostering violence, these values may also be employed to establish social hierarchies and consolidate relationships of domination in society. Our findings provide new insights into these dynamics and raise several important questions for future research.

## Data Availability

The datasets generated during the current study are available at the following link https://osf.io/dhcyt/.
